# *Escherichia coli* Morphological Changes and Lipid A Removal Induced by Reduced Pressure Nitrogen Afterglow Exposure

**DOI:** 10.1371/journal.pone.0116083

**Published:** 2015-04-02

**Authors:** Hayat Zerrouki, Virginie Rizzati, Corinne Bernis, Anne Nègre-Salvayre, Jean Philippe Sarrette, Sarah Cousty

**Affiliations:** 1 Université de Toulouse, UPS, INPT, LAPLACE (Laboratoire Plasma et Conversion d’Energie), Bât. 3R2, F-31062, Toulouse, France; 2 CNRS, LAPLACE, F-31062 Toulouse, France; 3 INSERM UMR 1048, University of Toulouse, Toulouse, France; 4 Université de Toulouse, UPS, Faculté de Chirurgie Dentaire de Toulouse, Centre Hospitalier Universitaire de Toulouse, F-31062, Toulouse, France; University of Malaya, MALAYSIA

## Abstract

Lipid A is a major hydrophobic component of lipopolysaccharides (endotoxin) present in the membrane of most Gram-negative bacteria, and the major responsible for the bioactivity and toxicity of the endotoxin. Previous studies have demonstrated that the late afterglow region of flowing post-discharges at reduced pressure (1-20 Torr) can be used for the sterilization of surfaces and of the reusable medical instrumentation. In the present paper, we show that the antibacterial activity of a pure nitrogen afterglow can essentially be attributed to the large concentrations of nitrogen atoms present in the treatment area and not to the UV radiation of the afterglow. In parallel, the time variation of the inactivation efficiency quantified by the log reduction of the initial *Escherichia coli* (*E*. *coli*) population is correlated with morphologic changes observed on the bacteria by scanning electron microscopy (SEM) for increasing afterglow exposure times. The effect of the afterglow exposure is also studied on pure lipid A and on lipid A extracted from exposed *E*. *coli* bacteria. We report that more than 60% of lipid A (pure or bacteria-extracted) are lost with the used operating conditions (nitrogen flow Q_N2_ = 1 standard liter per minute (slpm), pressure p = 5 Torr, microwave injected power P_MW_ = 200 W, exposure time: 40 minutes). The afterglow exposure also results in a reduction of the lipid A proinflammatory activity, assessed by the net decrease of the redox-sensitive NFκB transcription factor nuclear translocation in murine aortic endothelial cells stimulated with control *vs* afterglow-treated (pure and extracted) lipid A. Altogether these results point out the ability of reduced pressure nitrogen afterglows to neutralize the cytotoxic components in Gram-negative bacteria.

## Introduction

In 2005, a French senatorial report indicated that between 6 and 7% of the hospitalizations were complicated by a hospital-acquired infection, inducing more than 4.000 deaths per year [1–2]. Most of these infections were due to Gram-negative bacteria such as *Escherichia coli*, *Klebsiella pneumonia*, *Enterobacter* and *Salmonella* [3]. Recent epidemiologic studies have shown the presence of significant quantities of residual organic matter (up to 1.2 mg per instrument) at the surface of instruments after their treatment by sterile service departments [4–9]. Lipo-polysaccharides (LPS) and endotoxins molecules are among the most bioreactive residual bacterial agents, their presence in the bloodstream being involved in the release of pro-inflammatory mediators, with consequences ranging from mild fever to irreversible tissue injury, sepsis, and death [10–11]. LPS are constituents of Gram-negative bacteria membranes liberated in large amounts during bacterial death; they are insensitive to pH changes and extremely heat resistant, requiring temperatures of about 200–250°C during 30 to 60 minutes to be destroyed, much higher than the one used by conventional sterilization means [12–13].

As thermolabile materials are increasingly used in the manufacturing of reusable medical instrumentation, it appears crucial to rapidly conceive new sterilizing processes also able to remove LPS at low temperature. To this point of view, non-equilibrium plasma discharges are among the most promising techniques as they are able to produce large concentrations of physically and chemically reactive species at low temperature, using safe atmospheric gases. For medical instrumentation treatment, post discharge at reduced pressure (1–20 Torr) appear to be particularly interesting because of the increased diffusion of the active species, allowing to homogeneously treat large volumes (up to 10–20 liters) in absence of aggressive ionized species possibly inducing damages to the surface of the exposed instruments. Recently, the inactivation efficiency of a pure nitrogen afterglow was established, demonstrating the synergistic effect existing between the treatment temperature and the concentration of the nitrogen atoms present in the afterglow [14].

The present paper focuses on the interaction between active species produced by the pure N_2_ afterglow and bacteria (*E*. *coli*). In the first part, bacteria inactivation by the pure nitrogen post-discharge was evaluated by counting the number of colonies issued from surviving bacteria (colony forming units, CFU). New sets of experiments are presented in order to clarify the respective roles of the N-atoms and of the UV radiation in the inactivation processes of the nitrogen afterglow. The viability of the exposed bacteria was also checked via MTT tests. SEM observations of morphologic changes induced on *E*. *coli* by the N_2_ afterglow exposure are presented.

The second part of the paper is devoted to the removal of lipid A (a pyrogenic proinflammatory component of LPS, [15]) by the same nitrogen afterglow, with a particular focus on the inflammatory effect of the remaining by-products, characterized by the activation of the proinflammatory redox-sensitive NFκB transcription factor, a classical LPS target [16].

## Material and Methods

### Nitrogen afterglow and spectroscopy

**Fig 1:**
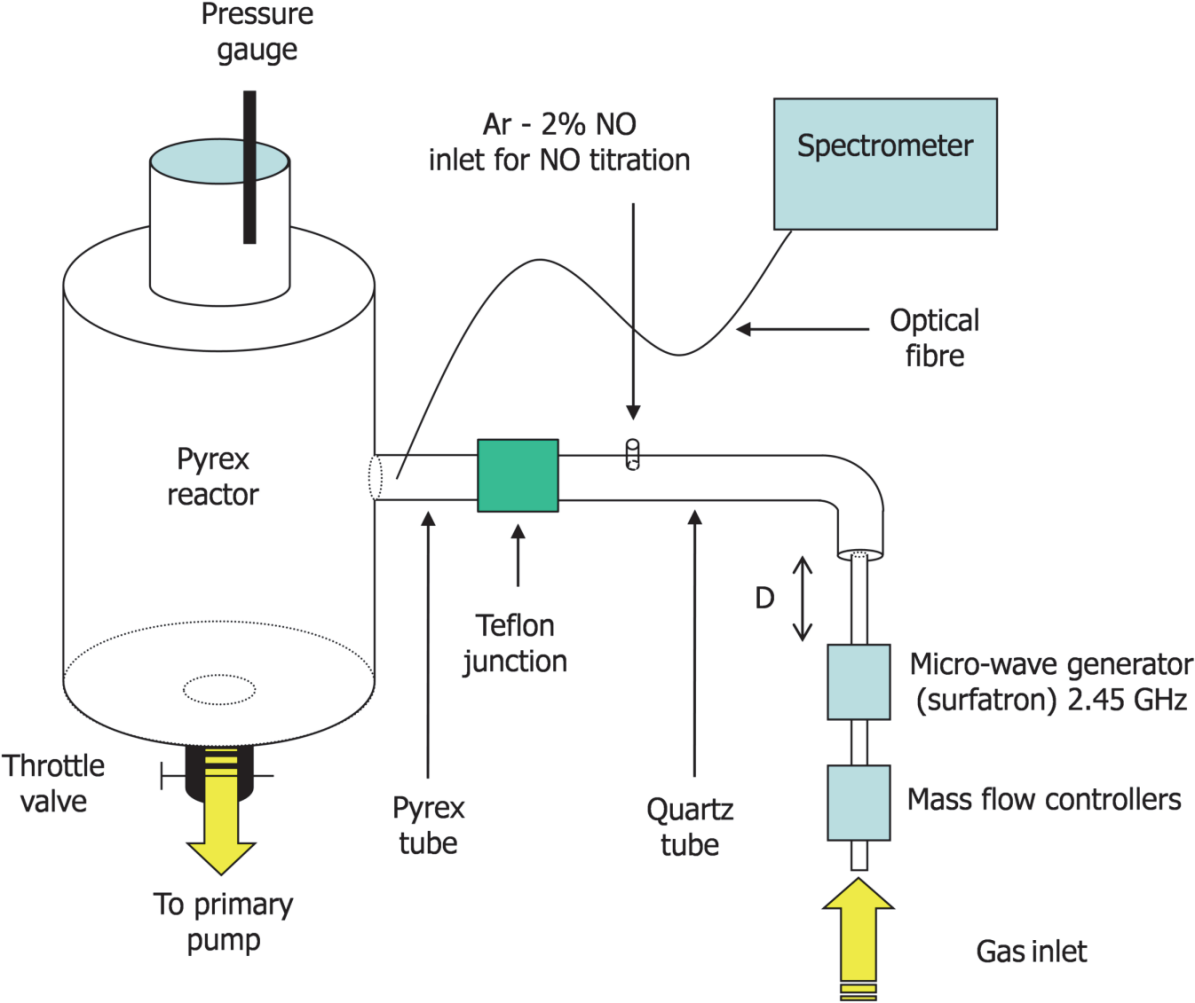
Flowing afterglow set up.

The used afterglow set up is presented in Fig. 1. It is composed of a surfatron cavity excited by a Sairem GMP 03 KE/D microwave generator operating at 2.45 GHz and producing a discharge in a quartz tube of internal diameter 4 mm at a power P_MW_ varying between 50 and 300 W. The N_2_ flow rate Q_N2_ is controlled by SLA 5850S Brooks flow-meters in the range between 1.0 and 3.0 slpm while the gas pressure p in the 5 litre cylindrical Pyrex reactor can be tuned between 4 and 30 Torr by means of a throttle valve. The 4 mm i.d. discharge tube enlarges to 19 mm before a bent and is connected by a Teflon junction to a Pyrex tube of identical diameter (19 mm) before the entrance of a 5 litre reactor where samples can be exposed to the afterglow flow. The total distance d between the surfatron and the reactor is set to 56 cm.

The operating parameters (p = 5 Torr, Q_N2_ = 1.0 slpm, P_MW_ = 200 W, D = 56 cm) were chosen in order to obtain pure late afterglow conditions (PLAC) in the treatment reactor. They are identical to the one used in previous inactivation experiments by Villeger et al. With such conditions, a gas temperature slightly higher than the room temperature (T = 30°C) and a high concentration of N atoms ([N] = 1.7 10^21^ m^-3^) were measured in the treatment chamber, using optical emission spectroscopy [14]. PLAC in the treatment region (reactor) insure a total absence of electrons and ions and a low N_2_(A) metastable density, less than 2 10^16^ m^-3^ [17].

Emission spectroscopy measurements were performed with a mobile optical fiber connected to an Acton Spectra Pro 2500i spectrometer (focal length 50 cm, grating 600 gr/mm) equipped with a front illuminated Pixis 256E CCD detector (1024 x 256 pixels).

To check the role of the UV irradiation in the inactivation mechanisms of the nitrogen late afterglow, a MgF_2_ filter was placed as a lid on the open Petri dish containing the bacterial film. With a cutting wavelength at 120 nm, the MgF_2_ filter allows the UV radiation at 320 nm to interact with the bacteria, while considerably reducing the access of the active species of the afterglow.

### Microbiological protocol

#### Colony forming unit counting

*Escherichia coli* (*E*. *coli*, CIP: 54.8 T) strain provided by the International Collection of the Pasteur Institute was used. After an overnight incubation in a Luria-Bertani (LB) broth at 37°C, bacteria were separated from the broth by centrifugation (10 minutes, 4000 g) and immersed in pure distilled water. A 10 μl droplet containing an average bacteria concentration of 10^7^–10^8^ / ml was deposited on a sterile glass sheet, placed in the treatment chamber in an open Petri dish and slowly desiccated by a vacuum exposure (10 minutes at 0.01 Torr), resulting in a bacterial film of 5–10 mm in diameter. The discharge was then turned on and the bacteria submitted to the pure nitrogen afterglow.

After exposure, the glass sheet and the bacterial film containing living and inactivated bacteria were immersed in 1 ml of LB broth. Bacteria were then retrieved by a 90 s gentle mechanical agitation. 100 μl of the recovery suspension were taken, eventually diluted and spread on agar in Petri dishes. The colonies formed from the surviving bacteria were finally numbered after 24 h of incubation at 37°C. For all the experiments, a control sample was made with no afterglow treatment (discharge off) and used as a reference.

The glass sheet and the exposed bacterial film were metallized prior to SEM observation (FEI Quanta 250 FEG).

#### Bacteria viability

The residual viability of the *E*. *coli* bacteria submitted to the N_2_ afterglow was estimated using the MTT [3-(4,5-dimethylthiazol-2-yl)-2,5-diphenyltetrazolium bromide] assay [18], immediately after the exposure. Bacteria recovered in 1 ml of LB broth were incubated for 3 h with the MTT solution (5 mg/ml) at a final concentration of 0.5 mg/ml. At the end, the cell suspension was centrifuged (2000 rpm, 5 min). The blue formazan crystals formed from reduction of MTT by living bacteria were dissolved in 200 μl of dimethylsulfoxide and the optical density was measured on a microplaque (540 nm, TECAN spectrophotometer GENios).

Alternatively, bacteria treated by low pressure alone (LP) or low pressure and late nitrogen afterglow (AG) were stained with the fluorescent nucleic acid probe DAPI. Briefly, slides recovered with bacteria were fixed by paraformaldehyde 4% in PBS, and stained with DAPI (0.1 mg/ml PBS), for 10 min. at room temperature. Slides were washed twice in deionized water and photographed by fluorescence microscopy.

### Lipid A quantification

A solution of lipid A (diphosphoryl from *Salmonella* Re 595 Minnesota, L0774 Sigma) was prepared by sonication (1 mg/ml) in H_2_O and was used as standard for quantification experiments. For this purpose, 1 μg of pure lipid A was desiccated on a sterile glass slide, resulting in a thin film of 5 mm in diameter, and was subjected during 40 minutes to the pure nitrogen afterglow, in the same conditions as bacteria (see in 2.2). At the end, lipid A was eluted from the microscope slides by 500 μl of a chloroform/methanol/water solution (73.3:23.3:3.3 respectively) twice. The chloroformic phase was dried under nitrogen, suspended in 20 μl of solvent mix, bath sonicated for 5 minutes and spotted on a nitrocellulose membrane. Membranes were blocked with a Tris-Nacl solution, containing 0.1% Tween 20 (TBST), 10% non fat dry milk, for 1 h at room temperature. Then, membranes were incubated overnight at 4°C with an anti-lipid A goat primary antibody (NB-600-1505 Novus France, Interchim, Montluçon France) using a dilution of 1/400 in 1% non fat milk TBST. A second incubation was done with an anti-goat immunoglobulin horseradish peroxydase coupled secondary antibody at a dilution of 1/5000 for 1 h at room temperature. After several washes, dots were detected using western blotting detection reagents (ECL, Amersham). Relative intensity of each spot was scanned and quantified using Image J software. Controls without afterglow exposure were done in the same conditions. The relative lipid A content (vacuum-treated and vacuum + afterglow exposed) was evaluated *vs* a dot-blot calibration curve of different lipid A concentrations ranging from 0.25 to 2 μg.

Alternatively, the lipid A content of control and afterglow-treated *E*. *coli* bacteria was estimated in the following conditions: approximatively 10 μl of bacteria suspension (average *E*. *coli* concentration of 10^7^-10^8^/ml) were spotted on glass slides and exposed to the afterglow, as described in 2.2. Bacteria were collected in water and lipid A was extracted by addition of a chloroform/methanol/H2O mix (73.3:23.3:3.3, v/v/v). As previously described for native lipid A, the solvent phase of samples were evaporated under nitrogen, suspended in 100 μl of mix and bath sonicated for 5 minutes. An aliquot of 5 μl was spotted on nitrocellulose membrane and the lipid A content was detected by Dot-blot, as above indicated.

### Nuclear translocation of the NF-κB transcription factor

The inflammatory effect of lipid A was investigated in murine aortic endothelial cells (CRL2181, American Type Culture Collection, Manassas, VA). Cells were grown in 100 mm culture dishes, in DMEM containing Glutamax and supplemented with 10% fetal calf serum (FCS), penicillin (100 units/mL) and streptomycin (100 μg/mL), (Invitrogen Cergy-Pontoise, France), as previously used in [19]. At sub-confluency, the medium was removed and replaced by fresh FCS free-DMEM medium. After 24 h, the cells were stimulated for 20 min by lipid A (200 ng/ml), untreated or vacuum-treated, and by an identical volume of afterglow-treated residual lipid A, in 0.5% FCS DMEM medium. A positive control was done using TNF-α (20 ng/ml, 20 min). In order to test the change of proinflammatory potential of bacteria, vacuum or afterglow-treated *E*. *coli* bacteria were collected in 100 μl of water and bath sonicated for 10 minutes. Then 10 μl of bacteria homogenates were incubated with cells for 2 hours.

At the end, the cells were washed 3 times in phosphate-buffered saline and the nuclei (containing the activated NF-κB transcription factor) were extracted using the NE-PER Nuclear and Cytoplasmic Extraction Reagents kit (Pierce), according to the manufacturer's protocol. Protein concentration in the purified nuclei was determined using the Bradford reagent (Biorad).

Equal amount (25 μg) of nuclei extracts were loaded on a SDS-polyacarylamide gel and electrotransferred to polyvinylidene fluoride membrane, under the previously used conditions [19]. Immunoblotting was performed with a primary anti-NFkappa B p65 antibody (ab16502 Abcam, Paris France) at the concentration of 0.5 μg/ml.

## Results and Discussion

### Bacteria inactivation by the nitrogen afterglow

#### Effect of the UV irradiation

It is well documented that the bacterial DNA can be highly damaged by absorption of radiation around 250 nm, conducing to a rapid cell death [20–23]. In our treatment chamber, a low UV intensity hardly distinguishable from the background signal of the acquisition system can be observed at 320 nm, corresponding to the NO β system emission (the NO γ emission was not observed). The NO β emission is due to the oxygen impurities contained in the nitrogen tank (Linde, HiQ Nitrogen 4.5, maximum impurity level less than 0.005%), dissociated in the discharge and recombining in the afterglow (reactions 1 and 2):

$$\text{N}+\text{O}+{\text{N}}_{2}\to \text{NO}\left(\text{B, v’=0}\right)+{\text{N}}_{2}$$ (1)

$$\text{NO}\left(\text{B, v’=0}\right)\to \text{NO}\left(\text{X, v’’=8}\right)+{\text{hv}}_{\text{320nm}}$$ (2)

For the used operating conditions, the NO β measured intensity at 320 nm is equal to 1% of the nitrogen first positive (1+) system intensity observed at 580 nm and coming from the N atoms recombination (reactions 3 and 4):

$${\text{N + N + N}}_{\text{2}}\to {\text{N}}_{\text{2}}\left(\text{B, v’=11}\right)+{\text{N}}_{2}$$ (3)

$${\text{N}}_{\text{2}}\;\left(\text{B, v’=11}\right)\to {\text{N}}_{\text{2}}\left(\text{A, v’’=7}\right)+{\text{hv}}_{\text{580nm}}$$ (4)

The band intensity I(λ) measured at a given wavelength λ depends of the concentration of the emitting state [N*] through the equation:

$$I\left(\lambda \right)=c\left(\lambda \right)\frac{hc}{\lambda }\left[N^{*}\right]A_{l}^{u}$$ (5)

where c(λ) is the spectral response of the measurement system, h and c are respectively the Planck constant and the light velocity and ${\text{A}}_{\text{l}}^{\text{u}}$ the vibrational transition probability of the observed band. In PLAC, as recently shown by Zerrouki et al. [17], the [O]/[N] density ratio can be deduced from the measured intensity ratio I(320nm)/I(580nm):

$$\frac{\left[O\right]}{\left[N\right]}=\frac{c\left(580\;nm\right)}{c\left(320\;nm\right)}\;K\;\frac{I\left(320\;nm\right)}{I\left(580\;nm\right)}\;,$$ (6)

with

$$K=0.55\;\frac{A_{N_{2}\;1+}^{11-7}}{A_{NO\;\beta }^{0-8}}\;\frac{k_{3}\left(\upsilon_{NO}^{R}+\left[N_{2}\right]k_{NO}^{Q}\right)}{k_{1}\left(\upsilon_{N_{2}}^{R}+\left[N_{2}\right]k_{N_{2}}^{Q}\right)}$$ (7)

In this expression, k_1_ and k_3_ are respectively the rate coefficients for reactions (1) and (3), while ν^R^ and k^Q^ are the radiative desexcitation frequency and the quenching rate coefficient of the emitting state. Using the data given in ref. [17] and for the 5 Torr pressure used in the present work ([N_2_] = 1.6 10^17^ cm^-3^), the calculated K value is 0.9. A calibration of the spectroscopic acquisition system was performed with a tungsten ribbon and the c(580nm)/c(320nm) ratio was found to be 5.7, corresponding to a [O]/[N] density ratio equal to 5%. With this low O-atoms concentration, the bactericidal effect of the nitrogen afterglow exposure can be attributed either to the N-atoms or to the UV production.

**Fig 2:**
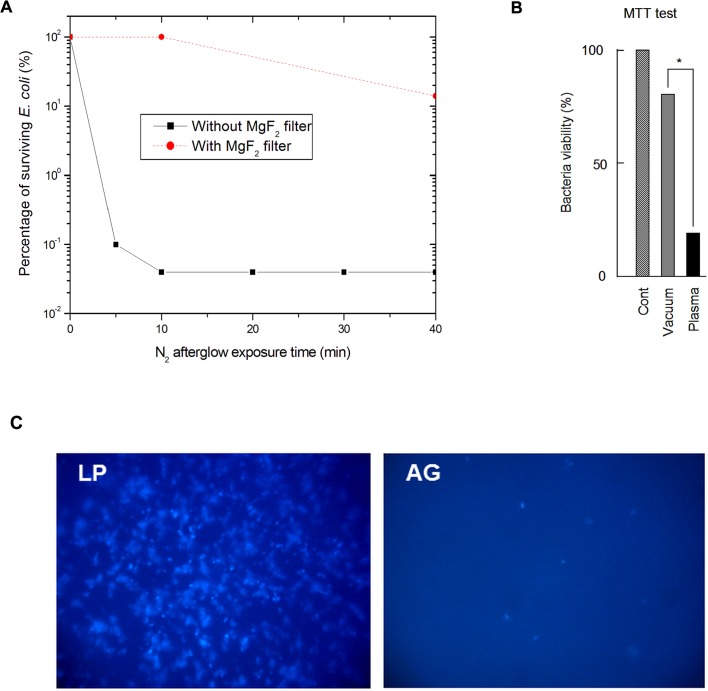
Effect of nitrogen afterglow exposure on bacteria inactivation and viability. A: Survival curves obtained for *E*. *coli* bacteria exposed to the nitrogen late afterglow with and without the MgF_2_ filter. B: MTT test in bacteria control (vacuum-treated) *vs* bacteria exposed to the nitrogen late afterglow. Bacteria were eluted from the slides with 1 ml of Broth medium, and incubated in this medium with the MTT reagent (5 mg/ml). After 3 h incubation at 37°C, the bacteria suspension was centrifuged, and the violet formazan crystals were dissolved in 200 μl of DMSO. The optical density was estimated on a microplaque TECAN reader. C. Pictures of bacteria submitted either to vacuum alone for 15 min (LP), or to vacuum + nitrogen late afterglow (AG), and stained with the nucleic acid fluorescent probe DAPI. The results are expressed as % of the vacuum-treated control. Mean +/-SEM of 5 separate experiments, * < p.0.05.

The survival curves obtained from CFU counting, with and without the MgF_2_ filter are shown in Fig. 2A. Results are expressed as % of inactivated bacteria, the reference (100%) corresponding to bacteria exposed to the 5 Torr N_2_ flux, discharge off.

No significant inactivation is found after a 10-minute exposure to the nitrogen afterglow when using the MgF_2_ filter. A 0.9 log reduction of the initial *E*. *coli* population is obtained after a 40-minute exposure. This reduction is much lower than the one obtained without the filter regardless of the exposure time (Fig. 2A). The NO β radiation is produced in the entire volume of the reactor and can reach the bacteria at the condition not to be reabsorbed. For a N_2_-2%O_2_ mixture (a mixture much richer in oxygen than the one used in the present work), it was shown by modelling by Kutasi that the order of magnitude of the NO(X) concentration present in the afterglow region was around 1 ppm [24]. For a N_2_-0.2%O_2_ mixture and similar afterglow conditions, a NO(X) density less than 5 10^9^ cm^-3^ was calculated by Pintassilgo [25]. With such density, the reabsorption probability of the NO β radiation is very low and UV photons emitted in the entire volume of the reactor are able to interact with the bacteria. As this volume is only reduced by 2% by the presence of the MgF_2_ filter, it demonstrates that the UV interaction with the bacterial DNA is not the major inactivation mechanism of the nitrogen afterglow.

It is to note that these results are completely different than the ones obtained by Boudam for *B*. *atrophaeus* spores exposed through a CaF_2_ filter (having a cutting wavelength at 112 nm) to a N_2_-0.3%O_2_ flowing afterglow, with a protocol very similar to the one used in the present work [22]. With the N_2_-0.3%O_2_ mixture used in Boudam’s work, no noticeable difference was found between the inactivation curves obtained with and without filter. In a recent paper by the same team [26], Moisan assumes that the main sterilization agent in N_2_/O_2_ afterglows are the UV photons emitted by the NO γ system between 205 and 280 nm, and produced by the alternative recombination scheme:

$$\text{N}+\text{O}+\left({\text{N}}_{\text{2}}\right)\to \text{NO}\left(\text{A}\right)+{\text{N}}_{2}$$ (8)

$$\text{NO}\left(\text{A}\right)\to \text{NO}\left(\text{X}\right)+{\text{hv}}_{\text{gamma}}$$ (9)

As the UV production due to the NO γ emission is maximal for the N_2_-0.3%O_2_ mixture [22] and was not observable in our experiments in pure N_2_, it strongly suggests that the bacterial inactivation mechanisms of the N_2_-0.3%O_2_ and of the N_2_ afterglows could be completely different.

#### Bacteria viability

The used classical colony counting method checks the ability of the *E*. *Coli* bacteria to grow and develop colonies after being exposed to the nitrogen afterglow. Nevertheless, when submitted to an environmental stress, bacteria can enter a viable but non culturable state (VBNC) [27–28]. In this particular state, they are no longer able to form colonies but still possess a reduced metabolic activity allowing them in some cases to restore their colony forming capability [29–30]. As the MTT test measures the dehydrogenase enzyme activity (which is necessary for the reduction of the MTT reagent), it can be used to distinguish between inactivated and VBNC bacteria.

The viability of the *E*. *coli* bacteria exposed to the nitrogen afterglow was estimated by the MTT test after 20 minutes of exposure (p = 5 Torr, Q_N2_ = 1.0 slpm, P_MW_ = 200 W). In these conditions and as shown in Fig. 2B, the residual viability observed for nitrogen afterglow-treated bacteria is close to 20%. This residual viability is significantly different from the high inactivation rate (higher than 99%, Fig. 2A) obtained with the colony formation test after exposure to the afterglow. The same discrepancy between viable and cultivable bacteria was recently observed by Dolezalova for *E*. *coli* exposed to an atmospheric pressure plasma jet [31].

This apparent discrepancy may result from the fact that the MTT test was performed immediately after the afterglow treatment, on bacteria still expressing enzymatic activity, whereas the colony test only quantifies surviving bacteria 24 h after the end of the afterglow treatment. However, MTT reduction activity in *E*. *coli* generally reflects the efficiency of the cellular electron transport, i.e. viable cells [32]. The observed discrepancy can also be attributed to the fact that cellular electron transport mechanisms are less impacted than others (growing mechanisms for example) by the N atoms flux of the afterglow.

Lastly, the strong interaction between the nitrogen afterglow and the bacteria was clearly evidenced by the use of DAPI, a fluorescent probe specific for nucleic acids, which points out the lack of fluorescence signal on slides submitted to the nitrogen afterglow (Fig. 2C AG) compared to low pressure controls (Fig. 2C LP). This result can be attributed either to a complete elimination of the nucleic acids (and of most of cytoplasmic components, as confirmed by the observed morphologic changes, see 3.1.3) or at least to their strong degradation, inhibiting the fixation of the fluorescent stain on nucleic acids.

#### Observed morphologic changes and inactivation scenario of the nitrogen afterglow

In oxygen containing flowing afterglows at reduced pressure, the admitted scenario of bacterial inactivation was initially proposed by Philip et al. [23]. It is based on a coupled effect between the chemically reactive species of the afterglow, mainly the atomic oxygen, eroding the external membrane and metabolic components of the bacteria, and the UV photons penetrating through the membrane to cause irreversible damages to the DNA strands [33].

In pure nitrogen afterglows, as stated in 3.1.1, the UV intensity is much lower than in O_2_ containing afterglow. Nevertheless, sterilization (considered here as a 6 log reduction of an initial bacteria population) can be obtained for exposure times of about one hour at low temperature [14,21,22] for both afterglows. As a consequence, a mechanism alternative to the UV action has to take place in oxygen free afterglows.

**Fig 3:**
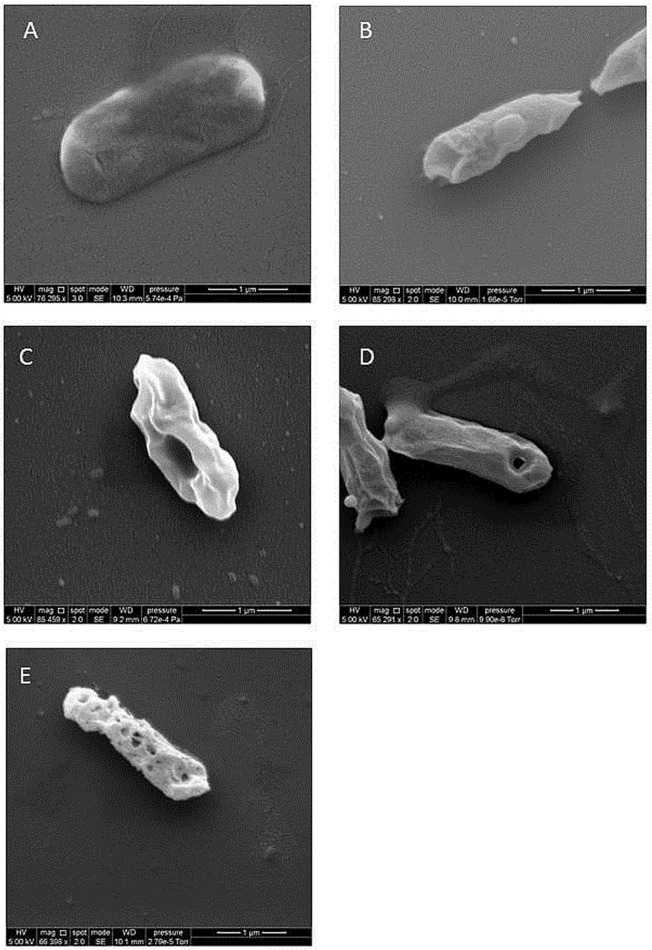
Effect of nitrogen afterglow exposure on bacteria morphology. A: Control (*E*. *coli*). B, C, D, E: After respectively 5, 10, 20 and 40 minutes of exposure to the pure N_2_ afterglow.

Fig. 3 shows SEM micrographs of *E*. *coli* exposed to the pure N_2_ afterglow for exposure times between 5 and 40 minutes. Compared to the control micrograph (Fig. 3A), the external roughness of the bacteria increases with the exposure time: after a 5 min. exposure (Fig. 3B), small wrinkles are clearly visible; after 10 min. (Fig. 3C) a large invagination is seen; after 20 min. (Fig. 3D) a hole appears and after 40 min. (Fig. 3E) the whole surface is riddled. In *E*. *coli*, the Gram-negative envelope is constituted by the plasma membrane and the cell wall (outer membrane, peptidoglycan layer, and periplasm) [34]. The outer membrane is porous to nutrients and other substances vital for the bacteria livelihood, but any increase in porosity may result in a loss of vital periplasmic constituents and modifications of periplasm osmolarity, leading to bacteria death. Based on these observations, the inactivation scenario evoked by the nitrogen afterglow could be the following:

1. continuous chemical etching by the nitrogen atoms of the afterglow inducing nanoscale damages of the cell wall and increasing its porosity; then damage to the cell membrane. After alteration of the cell wall, damage to the cell membrane must also be related to low pressure;
2. continuous extraction of metabolic components through increasing size holes, due to the vacuum action;
3. cell death when an irreversible stage is reached.

### Lipid A removal

Lipid A is a potent proinflammatory agent and the major responsible of the bioactivity and toxicity of endotoxin (LPS) in Gram-negative bacteria [15,35,36]. Lipid A is recognized by the Toll-like receptor 4 (TLR4) on host cells [16,36], which initiates a signaling cascade resulting in the activation of the redox-sensitive transcription factor NFκB, which plays a key role in regulating the immune response to infection. At the basal state, NFκB is maintained inactive in the cytosol by its cytoplasmic inhibitor IkB. Once activated, NFκB translocates into the nucleus where it binds DNA and stimulates the production of pro-inflammatory cytokines such as TNF-α and IL-6 [36].

**Fig 4:**
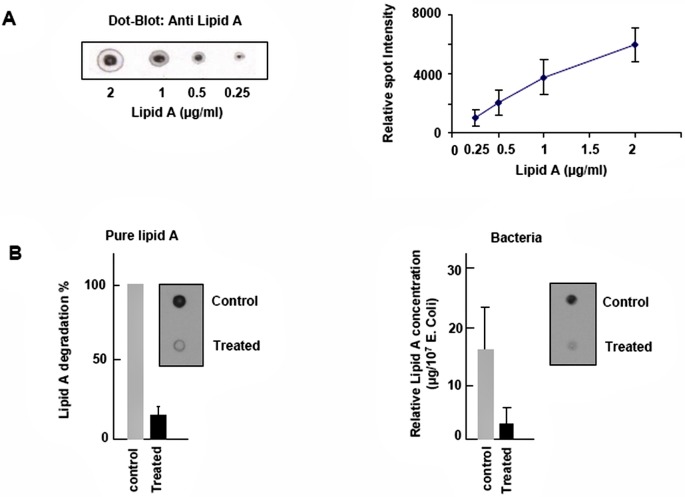
Effect of nitrogen afterglow exposure on lipid A. A. Dot blot binding assay: Increasing concentrations of lipid A were spotted on nitrocellulose membranes and blotted with an anti lipid A antibody. The relative intensity of each spot was quantified (Image J), allowing to build a dose-response calibration curve.B. Dot blots of lipid A pure (left panel) and present in *E*. *coli* extracts (right picture): 1 μg pure lipid A was spread off on sterile glass slides, and exposed to vacuum (control), or vacuum + nitrogen afterglow, in the conditions described in the Method section. At the end, the lipid A was eluted, spot on nitrocellulose membrane and immunoblotted with an anti lipid A antibody. The results are expressed as % of residual lipid A *vs* the vacuum-treated control. On the right panel, determination of the lipid A content in exposed bacteria. 10 μl of a bacterial solution (10^8^/ml), were spotted on glass slides and were treated with plasma. Bacteria extracts were collected, lysed and detected by dot blot for lipid A content. Dot blot results were analyzed with the dot calibration curve and relative quantity of bacteria lipid A estimated. In insert, pictures of lipid A dot-blots pure (left) or from bacteria (right), in vacuum-treated and vacuum + nitrogen afterglow treated conditions. Mean +/-SEM of 5 separate experiments, * < p.0.05.

We aimed at determining whether the afterglow exposure affects the lipid A content, in the exposed bacteria. For this purpose, we developed a dot blot method based on an immuno detection of lipid A. The specific binding of primary antibody was revealed by the binding of a second antibody engineered against the first one and coupled with a horseradish peroxydase. Spots were detected using enhanced chemiluminescence reaction. We first checked the effect of nitrogen afterglow on a pure lipid A film spread on sterile glass slides (1μg), that was treated similarly to the bacteria film. The residual lipid A content was quantified using a standard lipid A curve (Fig. 4A) and compared to 1μg lipid A treated by vacuum (but not by nitrogen afterglow). As shown in Fig. 4B, the residual lipid A present on the slide after exposure to the nitrogen afterglow was less than 20% of the control, indicating that the afterglow treatment allowed to remove or degrade the endotoxin from the treated slide. In *E*. *coli*, lipid A is located in the external membrane which is the outermost layer of the Gram-negative wall which separates the external environment from the periplasm [34]. Since SEM and viability results indicate a strong alteration of the *E*. *coli* structure upon treatment with the nitrogen afterglow, we expected that this treatment should affect the lipid A content. As shown on Fig. 4, a net decrease in lipid A content was observed in bacteria treated by the nitrogen afterglow, leading to the conclusion that lipid A was submitted to afterglow transformation.

**Fig 5:**
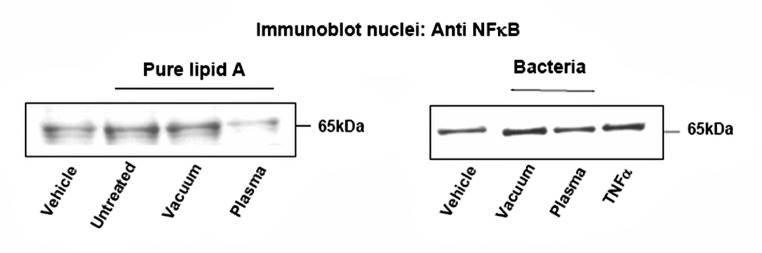
Nuclear translocation of the NFκB transcription factor. On the left, effect of pure lipid A: CRL2181 murine endothelial cells were treated for 20 min with lipid A (200 ng/ml) after low pressure treatment (vacuum), plasma treatment (5 Torr, 200 W, 40 min) (plasma) or from stock solution (untreated). A negative control without lipid A treatment was done (vehicle). At the end, cells were washed, the nuclei were extracted and used for SDS-PAGE electrophoresis and immunoblotting, using an anti NFκB antibody.On the right, effect of lipid A from bacteria: CRL2181 were stimulated for 2 h with 10 μl of *E*. *coli* extracts obtained after treatment with low pressure (5 Torr) for 15 min and N_2_ post-discharge at 200 W for 40 min, or low pressure only, for bacteria control. The nuclei were extracted and used for immunoblotting of NFκB as for pure lipid A.

We then investigated whether the afterglow exposure affects the proinflammatory properties evoked by lipid A, and assessed by the nuclear translocation of the NFκB transcription factor [16,35,36]. As shown in Fig. 5, 2 h treatment of the murine aortic endothelial cells CRL2181, by pure untreated lipid A (0.2 μg/ml) resulted in the nuclear translocation of NFκB, as reported [36]. Same results were obtained in the presence of vacuum-treated lipid A. In contrast, no nuclear translocation of NFκB was observed in cells stimulated by a same volume of afterglow-exposed lipid A. Since the proinflammatory and toxic activity of Gram-negative bacteria such as *E*. *coli* resides mainly in LPS, and more precisely in lipid A [36], we checked whether afterglow-treated bacteria may trigger the nuclear translocation of NFκB, by comparison with control and vacuum-treated bacteria. As shown in Fig. 5, bacteria extracts stimulated the translocation of NFκB, whereas nitrogen afterglow exposure inhibited this cellular response, indicating that the treatment by the afterglow strongly affects the biological activity of lipid A, probably by removing it from the support.

A positive control was done by stimulating CRL 2181 with TNFα (20 ng/ml, 20 min).These results are representative of 3 separate experiments.

## Conclusion

It is here shown that a pure nitrogen afterglow exposure strongly affects *E*. *coli* viability and substantially removes lipid A, the main proinflammatory and toxic component of LPS, the Gram-negative endotoxin.

As demonstrated with MgF_2_ experiments, the *E*. *coli* viability reduction is only correlated with the N atom concentration of the late afterglow and not with the UV-C production. In consequence, the cell death mechanisms are certainly different in pure N_2_ and in N_2_/O_2_ reduced pressure afterglows. In pure N_2_, due to the low UV intensity and the low etching rate of the N atoms, cell death appears to be due to a modification of the porosity of the cell wall and of the cell membrane by the N atoms and a direct matter extraction, as deduced from SEM observations of the exposed bacteria.

Concerning pure lipid A, we report a 80% loss (1 μg deposited at a concentration of 1 mg/ml) for a 40 minutes exposure to the pure N_2_ afterglow, similar to the one obtained with lipid A extracted from exposed *E*. *coli*. We also observed a net decrease of the proinflammatory activity of the exposed lipid A, assessed by the nuclear translocation of the redox-sensitive transcription factor NFκB. This lipid A loss during the afterglow exposure is consistent with the cell death scenario based on matter extraction.

As a conclusion, this study confirms the interest of pure nitrogen afterglows as a sterilizing system able to substantially reduce the amount of endotoxins (representing the main cause of bacteria virulence, even persistent in dead bacteria) at the surface of the reusable medical instrumentation.
